# Epidemiology of *Vibrio parahaemolyticus* Outbreaks, Southern Chile

**DOI:** 10.3201/eid1502.071269

**Published:** 2009-02

**Authors:** Erika Harth, Luis Matsuda, Cristina Hernández, Maria L. Rioseco, Jaime Romero, Narjol González-Escalona, Jaime Martínez-Urtaza, Romilio T. Espejo

**Affiliations:** Universidad de Chile, Santiago, Chile (E. Harth, L. Matsuda, J. Romero, R.T. Espejo); Secretaría Regional Ministerial de Salud, Puerto Montt, Chile (C. Hernández); Hospital Regional de Puerto Montt, Puerto Montt (M.L. Rioseco); Food and Drug Administration, College Park, Maryland, USA (N. González-Escalona); Universidad de Santiago de Compostela, Santiago de Compostela, Spain (J. Martínez-Urtaza); 1 Current affiliation: Helmholtz Centre of Infection Research, Braunschweig, Germany.

**Keywords:** Vibrio parahaemolyticus, shellfish, Chile, outbreaks, lateral transfer, research

## Abstract

Disease outbreaks caused by *Vibrio parahaemolyticus* in Puerto Montt, Chile, began in 2004 and reached a peak in 2005 at 3,600 clinical cases. Until 2006, every analyzed case was caused by the serovar O3:K6 pandemic strain. In the summer of 2007, only 475 cases were reported; 73% corresponded to the pandemic strain. This decrease was associated with a change in serotype of many pandemic isolates to O3:K59 and the emergence of new clinical strains. One of these strains, associated with 11% of the cases, was genotypically different from the pandemic strain but contained genes that were identical to those found on its pathogenicity island. These findings suggest that pathogenicity-related genes were laterally transferred from the pandemic strain to one of the different *V. parahaemolyticus* groups comprising the diverse and shifting bacterial population in shellfish in this region.

## Abstract 2

One-sentence summary for table of contents: Outbreaks are decreasing and the O3:K6 pandemic strain is being replaced by a new serotype and new strains.

In 1998 in Antofagasta, Chile (23°39′S, 70°24′W), ≈300 human cases of infection with *Vibrio parahaemolyticus* caused by consumption of contaminated seafood were reported (*1*). Outbreaks have not been observed in this region since 1998. During 2004–2007, ≈7,000 cases were reported farther south in Puerto Montt (41°29′S, 72°24′W) (*2**–*5). However, outbreaks generally have been decreasing; there were ≈1,500 cases in 2004, 3,600 in 2005, 900 in 2006, and 475 in 2007 (*6*) (http://epi.minsal.cl/epi/html/Actualidad/Vibrio.htm).

Until 2006, ≈100% of the cases analyzed were caused by a clonal group originally observed in Southeast Asia in 1996 (*4*,*5*,*7*). This group was known as the *V*. *parahaemolyticus* pandemic strain because it had reached coastal environments worldwide and caused outbreaks. It belongs to the O3:K6 serovar, although at least 21 serovariants have emerged since 1996 (*8*). These serovariants also have specific sequences corresponding to genes such as *toxRS*/*new* (*9*), open reading frame 8 (*orf8*) (*10*), and *tdh*, but lack others such as *trh*, which is found in other pathogenic strains.

Genome sequencing of the RIMD2210633 pandemic strain showed that it has 2 sets of gene clusters that encode the type III secretion system (TTSS) apparatus (*11*). This apparatus is used by several gram-negative pathogenic bacteria to secrete and translocate virulence factor proteins into the cytosol of eukaryotic cells (*12*). TTSS1 is involved in cytotoxicity against HeLa cells and TTSS2 is involved in enterotoxic activity in a rabbit ileal loop test (*13*). The first cluster is located on the large chromosome, and the second is located on the small chromosome. The second cluster contains 2 copies of the *tdh* gene and is located on a pathogenicity island (a discrete genetic unit that contains genes responsible for pathogenicity and virulence) probably obtained by recent lateral transfer (*11*). TTSS2 has been found only in strains showing β-type hemolysis on a specialized blood agar medium known as Wagatsuma agar (*11*). This hemolysis is called the Kanagawa phenomenon and is considered a useful marker for identification of pathogenic strains. Recently, TTSS genes related to the TTSS2 cluster were reported in clinical and environmental non-O1, non-O139 *V*. *cholerae* strains (*14*).

The clonal nature of the pandemic *V*. *parahaemolyticus* isolates was ascertained by the similarity of patterns obtained by genome restriction fragment length polymorphism–pulsed-field gel electrophoresis (*15*), arbitrarily primed PCR (*7*,*9*), direct genome restriction enzyme analysis (DGREA) (*4*), and multilocus sequence typing (MLST) (*16*,*17*). However, the pandemic strain is a minor fraction of a diverse and shifting *V*. *parahaemolyticus* population found in shellfish in Puerto Montt (*2*). In an effort to understand the epidemiology of these outbreaks, we studied *V*. *parahaemolyticus* isolates obtained from human cases and shellfish during the summer of 2007. Our results indicate replacement of the O3:K6 pandemic strain by new serotype O3:K59 and new pathogenic *V*. *parahaemolyticus* groups. The decrease in the number of clinical cases may have been caused by diminution of the *V*. *parahaemolyticus* O3:K6 pandemic group in regional seafood.

## Methods

### Strains

*V*. *parahaemolyticus* RIMD 2210633 (VpKX) and RIMD 2210086 (VpI) were obtained from the Research Institute for Microbial Diseases, Osaka University, Osaka, Japan. The Chilean environmental strains, identified as PMA with a number according to origin and year of isolation, were obtained from shellfish samples obtained during outbreaks from 2004 though 2007. Most strains have been described (*4*). PMC38.7, PMC60.7, PMC53.7, and PMC75.7 are isolates from clinical samples obtained in 2007. Each of these isolates corresponds to the type isolate of the 23 groups differentiated by DGREA as described (*4*) and reported in this article.

### Analysis

Samples of clinical cases and shellfish were obtained and analyzed as described (*4*). Isolation, growth, and characterization of isolates, including their DGREA patterns, were conducted as described (*4*,*5*). Each DGREA pattern found in 2007 was compared with those for previous years. When similarities in patterns were observed, their identity was checked by comparing patterns obtained in the same electrophoretic analysis.

PCR assays were performed by using ≈10 ng of total bacterial DNA per reaction tube. Amplification conditions were those previously reported for *tlh*, *tdh*, and *trh* (*18*), orf8 (*19*), and *toxRS/new* (*9*) genes. T3SS2 genes (VPA1335, VPA1338, VPA1339, VPA1341, VPA1342, VPA1346, VPA1349, VPA1354, VPA1355, VPA1362, and VPA1367) were amplified by using primers reported for a microarray assay at 61ºC by Meador et al (*20*). Genes VPA1321 and VPA1376, which are located at the extremes of the pathogenicity island, were amplified by using primers designed using the Primer3 program (http://primer3.sourceforge.net). Sequences of these primers were VPA1321f: 5′-TGACATGCACGGCAATAGAT-3′, VPA1321r: 5-ACAGAGTTGGTTTCGCAGGT-3′, VPA1376 f: 5′-CATCGAGCGATCTTTCACAA-3′, and VPA1376r: 5′-ACCGGTTTCCAACCTTCTCT-3′. Housekeeping genes for MLST were amplified by using primers for *V*. *parahaemolyticus* (*17*) and from the MLST website (http://pubmlst.org/vparahaemolyticus) developed by Keith Jolley (University of Oxford, Oxford, UK) (*21*).

PCR products were purified by using either the Wizard SV Gel or PCR Clean-Up Systems (Promega, Madison, WI, USA) and sequenced in both directions by Macrogen (Seoul, South Korea) or by McLAB (South San Francisco, CA, USA) by using forward and reverse amplification primers or primers M13F and M13R (MLST loci). DNA sequences were analyzed individually and manually assembled. Alignments and sequence similarities were obtained by using BioEdit software (*22*). Sequences obtained were deposited in GenBank under accession nos. EU185060–EU185092.

Amplification and sequencing of the variable region of the 16S rRNA (*rrs*) between nucleotides 357 and 518 (*Escherichia coli* numbering) were performed as described (*23*). This analysis consisted of separation of *rrs* alleles in PMC38.7 by pulsed-field gel electrophoresis and PCR amplification of the variable region in excised bands as described (*24*).

## Results

### *V. parahaemolyticus* Associated with Human Cases in 2007

**Figure 1:**
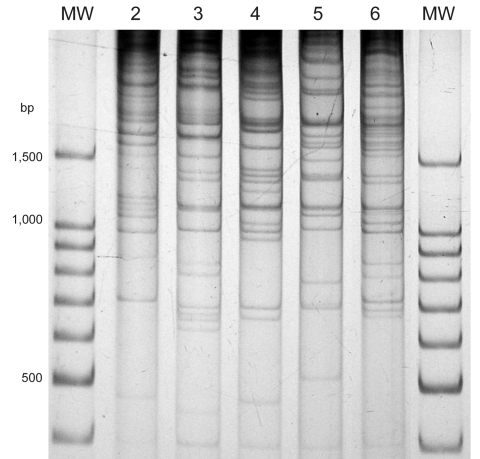
Direct genome restriction enzyme analysis with *Nae*I of clinical isolates of *Vibrio parahaemolyticus* representative of the 5 patterns observed during the outbreaks in Puerto Montt, Chile, January and February, 2007. Lanes MW, 100-bp size ladder; lane 2, PMC38.7; lane 3, PMC60.7; lane 4, PMC53.7; lane 5, PMC75.7; lane 6, VpKX. (O3:K6 pandemic isolate).

*V*. *parahaemolyticus* isolates from 37 human case-patients with diarrhea from the summer of 2007 in the Puerto Montt region were analyzed and grouped according to serotype, presence of genetic markers (*orf8*, *toxRS/new*, *tlh*, *tdh*, *trh)* and distinctiveness of their DGREA patterns (Table 1, Figure 1). One isolate from each patient was characterized. On the basis of genetic markers and DGREA pattern, isolates from 27 patients corresponded to the pandemic clonal group. However, 40% of the 20 serotyped strains of this group contained a K_59_ capsular antigen instead of the characteristic K_6_ antigen, and 25% cross-reacted with antisera for K_6_ and K_59_ antigens. Another difference from strains of previous years was that the relative number of cases associated with the pandemic strain (73%) was lower than the 100% observed in previous summers (*4*). Isolates from the other 27% of patients with clinical cases lacked the characteristic markers of the pandemic strains, i.e., *orf8* and *toxRS/new*, and had 4 new DGREA groups. One of these groups contained 4 *tdh-*positive isolates (11% of cases). A second group contained 4 isolates positive for *tdh* and *trh* genes. The other 2 groups contained 1 isolate each, and both were negative for these 2 markers (Table 1). One isolate chosen as the type strain of each group was typed by MLST (*17*). MLST sequence type corresponded with groups determined by other analyzed genetic properties (Table 1).

**Table 1:** Properties of *Vibrio parahaemolyticus* clinical isolates from Puerto Montt, Chile, summer 2007*

Isolate	*tlh*	*tdh*	*trh*	*orf8*	ToxRS/new	Serotype	DGREA group	MLST ST†
PMC50.7, 51.7, 41.7, 72.7, 1.7, 15.7, 16.7	+	+	–	+	+	O3:K6	VpKX	3
PMC55.7, 56.7, 58.7, 28.7, 44.7, 73.7, 14.7, 18.7	+	+	–	+	+	O3:K59	VpKX	ND
PMC29.7, 42.7, 70.7, 11.7, 19.7	+	+	–	+	+	O3:K6,59	VpKX	ND
PMC**59.7**, **63.7**, **64.7**, **65.7**, **66.7**, **20.7, 22.7**	+	+	–	+	+	ND	VpKX	ND
PMC38.7, **47.7**, 57.7, 68.7	+	+	–	–	–	O10:K20	38.7	63
PMC60.7, **25.7***,* **26.7**, 27.7	+	+	+	–	–	O1:KUT	60.7	64
PMC53.7	+	–	–	–	–	O3:K59	1.5	28
PMC75.7	+	–	–	–	–	O1:KUT	75.7	65

*Isolates with an undetermined serotype are in **boldface**. *orf*, open reading frame; DGREA, direct genome restriction enzyme analysis; MLST, multilocus sequence typing; ST, sequence type; ND, not determined. †Sequences are available from http://pubmlst.org/vparahaemolyticus.

### Characterization of Nonpandemic Strains

Strains positive for *tdh,* other than the pandemic strain, had not been isolated from patients with clinical cases in Puerto Montt during the 3 previous summer outbreaks. It has been reported that this gene may be spread by insertion sequence–like elements (*25*,*26*). The possibility that *tdh* found in nonpandemic strains was derived from the pandemic strain was explored. PCR amplicons of the *tdh* gene of the 2 nonpandemic groups was sequenced in isolates designated as type strains for each group: PMC60.7 for the group containing *tdh* and *trh* and PMC38.7 for the group containing only *tdh*. The amplicon of isolate PMC60.7 had an identical sequence to that reported for *tdhA* except for 1 nucleotide (*11*). Conversely, PMC38.7 had an identical sequence to that expected for a mixture of the 2 *tdh* genes in VpKX. These 2 genes differ slightly and the mixture of their PCR products should show polymorphisms in specific sites (*11*).

This observation suggested the presence of *tdhS* and *tdhA* genes in PMC38.7 with identical sequences to those found in the pandemic strain. Because these 2 genes are located close to each end of the pathogenicity island in chromosome 2 of the pandemic *V*. *parahaemolyticus* (*11*), the presence of the entire island was explored by PCR amplification of 11 genes of TTSS2 located in the island and of genes VPA1321 and VPA1376 located at the extremes of the island near *tdhA* and *tdhS*, respectively. Each tested gene was found in the PMC38.7 strain, and sequences of their PCR products were identical to those reported for the pandemic strain genes, except for 1 nucleotide in VPA1342. Serotyping of PMC38.7 indicated an O10:K20 serovar. Because MC38.7 is genetically different from the pandemic strain (Table 1, Figure 1), the high degree of homology of these genes suggested that the entire pathogenicity island had recently been transferred from the pandemic strain.

PMC38.7 also differs from the pandemic strain and most clinical isolates by the presence of intragenomic heterogeneity among its multiple 16S rRNA genes, a feature seldom observed in clinical isolates but frequently observed among environmental isolates. This finding is probably caused by lateral transfer of *rrs* (*23*). Three *rrs* genes, with sequences corresponding to *V*. *parahaemolyticus* groups VpD1-B4, ATA65-B2, and VpKX-AB (*23*), were observed in PMC38.7.

### *V*. *parahaemolyticus* Associated with Shellfish

**Figure 2:**
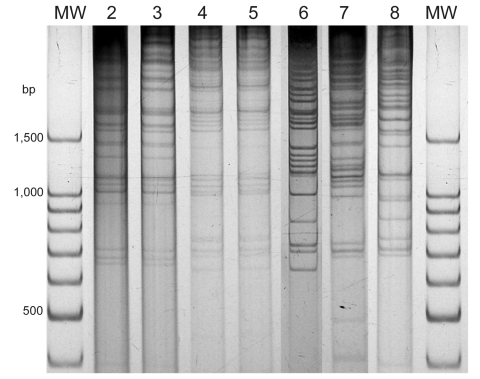
Direct genome restriction enzyme analysis with *Nae*I of *Vibrio parahaemolyticus* isolates from shellfish collected in Puerto Montt, Chile, summer, 2007. Gel shows representative strains for every observed pattern. Patterns of groups observed in previous years are next to the type isolate of that group. Lanes MW, 100-bp size ladder; lane 2, PMA4.7; lane 3, 34.6; lane 4, PMA9.7; lane 5, 118; lane 6, PMA1.7; lane 7, PMA11.7; lane 8, PMA21.7.

**Figure 3:**
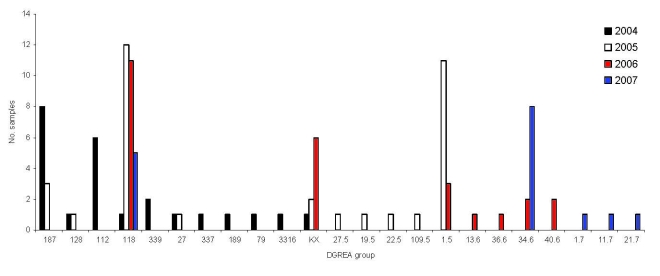
Number of seafood samples containing *Vibrio parahaemolyticus* corresponding to different direct genome restriction enzyme analysis (DGREA) groups observed in Puerto Montt, Chile, each summer, 2004–2007.

There are a large number of *V*. *parahaemolyticus* strains in the environment in the Puerto Montt region. Only the pandemic strain was isolated from clinical samples before and during the summer of 2006, but 20 different strains were isolated from shellfish during that period (*2*). Characterization of 52 isolates from 20 shellfish samples during the summer of 2007 indicated ≥5 DGREA groups; 3 of them were not previously observed (Table 2, Figure 2). This finding increases the number of different strains found in shellfish of the region to 23. Figure 3 shows the number of shellfish samples containing each of the 23 DGREA groups found during the past 4 years, including the summer of 2007 (*2* and this study). Only 4 of the 20 shellfish samples examined produced *tdh-*positive enrichment cultures, which is indicative of pathogenic strains. However, no *tdh*-positive isolates were obtained from these enrichments after plating on thiosulfate citrate bile salts sucrose agar.

**Table 2:** Properties of *Vibrio parahaemolyticus* isolates from shellfish from Puerto Montt, Chile, summer 2007*

Isolate	*tlh*	*tdh*	*trh*	*orf8*	ToxRS/new	DGREA group	MLST ST†
PMA4.7, 5.7, 6.7, 7.7, 8.7,15.7, 16.7, 17.7, 19.7, 20.7, 22.7, 23.7, 24.7, 25.7, 26.7, 27.7, 28.7, 33.7, 37.7, 41.7, 42.7, 43.7, 47.7, 48.7, 49.7	+	–	–	ND	ND	34.6	ND
PMA9.7, 10.7, 12.7, 14.7, 18.7, 38.7	+	–	–	ND	ND	118	10
PMA1.7, 2.7, 3.7	+	–	–	ND	ND	1.7	ND
PMA11.7, 13.7	+	–	–	ND	ND	11.7	ND
PMA21.7	+	–	–	ND	ND	21.7	ND

*orf, open reading frame; DGREA, direct genome restriction enzyme analysis; MLST, multilocus sequence typing; ST, sequence type; ND, not determined. †Sequences are available from http://pubmlst.org/vparahaemolyticus.

### T3SS2 Genes in Other Nonpandemic *V*. *parahaemolyticus* Strains

Because the presence of TTSS2 genes is not exclusive to the pandemic strain (*20*), their occurrence in the other environmental and clinical *V*. *parahaemolyticus* DGREA groups found in Puerto Montt was explored. We analyzed by PCR amplification 20 strains corresponding to 18 environmental DGREA groups (PMA79, 112, 118, 189, 337, 339, 3316, 1.5, 19.5, 22.5, 27.5, 13.6, 34.6, 36.6, 40.6, 1.7, 11.7, and 21.7) and 1 strain from each of 3 new clinical groups (PMC60.7, 53.7, and 75.7) found in Puerto Montt in 2007 for the VPA1335 gene (found in T3SS2). Among these strains, only PMA339, isolated from shellfish in the summer of 2004, was positive. PCR amplification of PMA339 for the other genes in the pathogenicity island showed positive reactions for all the genes tested in PMC38.7. Nevertheless, sequences of amplicons showed strong differences from those found in PMC38.7 and those reported for the pandemic strain genome. Similarity ranged from 99.4% for VPA1362 to 93.8% for VPA1346; the average for all genes tested was 97.7%. These differences indicate a much larger evolutionary distance between the T3SS2 genes in PMA339 and the pandemic strain than between PMC38.7 and the pandemic strain.

## Discussion

The epidemiology of outbreaks caused by *V*. *parahaemolyticus* in the Puerto Montt region is changing. The number of clinical cases caused by the pandemic strain has decreased, accompanied by a change of serotype from O3:K6 to O3:K59. The changing serotype of the pandemic strain has been recently reviewed by Nair et al (*8*). These authors showed that the more recent serotypes do not have the propensity for increasing hospital admissions observed with O3:K6, and some type of change in the epidemic process seems to be evident. Genes for the biosynthesis of capsular polysaccharides, which are major antigens (K) of *V*. *parahaemolyticus*, are probably encoded in a gene cluster characterized by variability that may occur through lateral gene transfer (*27*). Isolates reacting with antisera for K_6_ and K_59_ antigens may have changed part of the K epitopes reacting with the commercial polyclonal antiserum.

The percentage of clinical cases caused by the pandemic strain decreased from 100% in 2006 to 73% in 2007. Four clinical strains, not previously observed, emerged in 2007. Among these, 1 group representing 11% of the clinical cases, with type strain PMC38.7, may have recently received the genes on the pathogenicity island of the pandemic strains. Pathogenicity island genes identical to those in the pandemic strain in this bacterial group and differences in housekeeping genes and DGREA patterns are best explained by transfer of the pathogenicity island from the pandemic strain to an indigenous strain. The indigenous *V*. *parahaemolyticus* population in shellfish is diverse, and the predominant strains seem to change every year. A detailed examination of the putative genomic island in PMC38.7, its integration site, and its flanking regions, will probably help differentiate among possible mechanisms of DNA transfer.

The presence of TTSS2 genes is not exclusive of the pandemic and PMC38.7 strain; they were also found in an environmental isolate of *V*. *parahaemolyticus* (PMA339). However, PMA339 has not been observed among clinical isolates. TTSS2 genes have been found in other *V*. *parahaemolyticus* clinical strains (*20*); however, we identified them in environmental strains. Although on the basis of sequences obtained from their amplicons, TTSS2 genes in PMA339 seem to have independently evolved from the pandemic strain over a considerable time, they are still more closely related to the TTSS2 of *V*. *parahaemolyticus* than to those recently found in non-O1 or non-O139 *V*. *cholerae* (*14*).

Little is known of the origin of the other 3 clinical groups (60.7, 1.5, and 75.7). Group 60.7 contains *tdh* and *trh*, but this *tdh* does not seem to be derived from the pandemic strain. Strains of groups 1.5 and 75.7 lack both pathogenicity-associated genes, but finding these isolates in patients is not unusual (*28*). PMC75.7, a clinical strain, contains a *recA* gene that is closely related to that of PMA339, the environmental isolate containing TTSS2 genes (*17* and http://pubmlst.org/vparahaemolyticus). However, in view of a recent report (*29*), one should consider that the 2 human isolates lacking *tdh* or *trh* genes may correspond to nonvirulent strains that proliferate during infection with a virulent strain.

The abundance and frequency of pandemic and nonpandemic *V*. *parahaemolyticus* in shellfish seem to have been less in 2007 than in previous years. In 2006, 10 of 20 shellfish samples were positive for *tdh* after enrichment; in 2007, only 4 of 20 samples were positive. PCR amplification of colonies obtained after plating the enrichment culture on thiosulfate citrate bile salts sucrose agar enabled identification of *tdh-*positive colonies in 6 samples in 2006; none could be identified by the same method in 2007. The observed decrease in outbreaks was probably caused by a decrease in raw seafood consumption as a result of a public health campaign and a decrease in the load of the highly virulent pandemic strain in shellfish. However, this tendency could change on the basis of dispersion and virulence of emerging pathogenic strains.
